# The mechanism of action of micafungin against pteropine orthoreovirus infection in the human A549 cell line

**DOI:** 10.1007/s00705-025-06369-4

**Published:** 2025-08-25

**Authors:** Wirayatida Bubphasook, Atsuo Iida, Eiichi Hondo

**Affiliations:** Laboratory of Animal Morphology, Graduate School of Bioagricultural Sciences, Nagoya University, Nagoya, 464-8601 Japan

## Abstract

Pteropine orthoreovirus (PRV) is a fusogenic virus carried by bats that causes respiratory illnesses in humans. Micafungin (MCFG), an approved drug for treatment of fungal infections, has been shown to inhibit the propagation of PRV, but its precise mechanism of action remains unclear. In this study, we investigated the molecular mechanism of action of MCFG against PRV propagation. A molecular docking simulation showed that the p17 protein of PRV is likely to be the primary target of MCFG. Differential gene expression analysis was performed to compare MCFG-treated PRV-infected host cells with untreated infected cells, and IL-6 was found to be the main regulator induced by MCFG. Silencing of IL-6 using siRNA resulted in markedly increased levels of PRV release and syncytium formation and marginally increased PRV RNA replication. Treatment with an antibody against p17, the presumed target of MCFG, markedly reduced syncytium formation but did not influence viral RNA replication. In contrast, MCFG significantly suppressed syncytium formation and slightly reduced PRV RNA replication, and MCFG and anti-p17 antibody both increased IL-6 mRNA expression. Molecular docking analysis also suggested that MCFG might inhibit other PRV proteins, including the nonstructural replication protein σNS. In conclusion, it is likely that MCFG primarily targets p17 and modulates host immunity through IL-6, which probably interferes directly with syncytium formation.

**Supplementary Information:**

The online version contains supplementary material available at 10.1007/s00705-025-06369-4.

## Introduction

Many viruses, including severe acute respiratory syndrome coronavirus (SARS-CoV) [1], SARS-CoV-2 [2], Middle East respiratory syndrome coronavirus (MERS-CoV) [3], Ebola virus [4], and Nipah virus [5], are bat-borne pathogens of global significance. These viruses can cause severe illness in humans and result in substantial economic losses in the healthcare, agriculture, trade, and tourism sectors [6]. Preventing outbreaks and developing effective treatments are critical for reducing the effects of these diseases.

Pteropine orthoreovirus (PRV) is a fusogenic double-stranded RNA virus belonging to the family *Spinareoviridae* [7]. Its genome is made up of 10 double-stranded RNA (dsRNA) segments (L1-L3, M1-M3, and S1-S4). Some fusogenic reoviruses, such as avian orthoreovirus (ARV), broome orthoreovirus (BroV), baboon orthoreovirus (BRV), and PRV, encode two distinctive nonstructural proteins, the fusion-associated small transmembrane (FAST) protein, and p17, located on the S1 or S4 gene segment [8–11]. This virus was first reported in 2006, causing induced respiratory disease in humans, and has been isolated from patients in Malaysia, Indonesia, and the Philippines [12–16]. PRV causes acute respiratory infections (ARIs) [17] with a complex array of symptoms, including cough, sore throat, and high fever, with some individuals also experiencing diarrhea and vomiting [7]. So far, 15 strains of PRV have been isolated from bats of the species *Pteropus poliocephalus*, *P*. *homelands*, *P*. *vampyrus*, *Rousettus leschenaultia*, *R*. *amplexicaudatus*, and *Eonycteris spelaea* in Australia, Malaysia, Indonesia, China, and the Philippines [18–24]. The detection of PRV in fecal samples from monkeys in Thailand indicates its potential role in the transmission of PRV to humans [25]. PRV can also be transmitted from human to human, which could lead to outbreaks and epidemics if not detected and controlled early on [12, 26].

While viral outbreaks pose significant challenges, drug interventions offer promising solutions that can enhance control measures [27]. Drug repurposing seeks to identify new clinical uses for existing drugs with established safety profiles, pharmacokinetics, and other clinical characteristics, reducing the time and costs associated with developing new treatments [27, 28]. Micafungin (MCFG), an antifungal drug of the echinocandin class, is derived from fungal secondary metabolites and consists of a cyclic hexapeptide core with a lipid side chain [29]. MCFG specifically targets the fungal cell wall by disrupting proteins responsible for synthesizing β−1,3 glucan polysaccharides [30]. It has been approved by the United States Food and Drug Administration (US FDA) for treating *Candida* sp. infections [31], but it has also been shown to have to have antiviral activity against enterovirus 71 [32], chikungunya virus [33], dengue virus [34], and SARS-CoV-2 [35].

Previous work showed that MCFG exhibits antiviral activity against PRV infection in human cell lines by effectively inhibiting viral release and formation of syncytia [36]. However, the mechanism by which micafungin inhibits this virus remains unknown. In this study, we used molecular docking simulations to predict which PRV proteins might be targeted by MCFG, and next-generation sequencing to examine gene expression profiles to study the effect of the target PRV protein on host cell responses. A candidate gene was knocked down by siRNA to investigate its role in PRV RNA replication. Lastly, an antibody against the target protein of MCFG was generated to investigate the effect of MCFG through functional viral inhibition assays. Understanding the molecular mechanisms of MCFG is crucial for advancing antiviral therapy, ensuring the development of effective treatments, and preventing future outbreaks.

## Materials and methods

### Molecular docking

Amino acid sequences of PRV proteins were obtained from the NCBI database (https://www.ncbi.nlm.nih.gov) and are shown in Supplementary Information 1A. Viral protein structures were modeled using Alphafold2 (https://alphafold.ebi.ac.uk) [37]. The 2D structure of micafungin was obtained from the PubChem database (https://pubchem.ncbi.nlm.nih.gov), and its 3D structure was predicted and optimized using Avogadro (v1.2.0) [38]. Molecular docking experiments were conducted using AutoDock 4.2.6 (http://autodock.scripps.edu/) [39–41]. During the docking process, explicit hydrogens, charges, and flexible torsions were assigned to the protein and the ligands. Polar hydrogens were added to ensure the correct ionization and tautomeric states of the amino acid residues of the protein, and Kollman united atom charges were applied to the protein. The rigid roots of ligands were identified, with rotatable bonds set to allow flexibility. The modified 3D structures of the viral protein and ligands, which incorporated their bond flexibility, were converted to the PDBQT format required for AutoDock calculations. The Lamarckian genetic algorithm (LGA) was employed to explore potential binding conformations using the following settings: 300 dockings in the population, a maximum of 27,000 generations, 25 million energy evaluations, and 50 docking runs with randomized initial positions and conformations. Default parameters were applied for other settings, including mutation and crossover rates. AutoGrid was used to define the search space by generating a grid encompassing the entire protein structure. Pre-calculated grid maps representing interaction energies between ligand atom probes and the receptor were generated with AutoGrid 4.2. The conformation with the lowest binding energy was selected as the most favorable binding pose. All docking results, including images and outputs from AutoDock, were further analyzed using UCSF Chimera (https://www.cgl.ucsf.edu/chimera/) [42].

### Cells

Vero JCRB9013 (African green monkey kidney cells) and A549 JCRB0076 (human lung tissue cells) were used in this study. Both cell lines were cultured and maintained in Dulbecco’s modified Eagle’s medium (DMEM; Nissui, Tokyo, Japan) containing 10% heat-inactivated fetal bovine serum (FBS; HyClone, Logan, UT), 2 mM L-glutamine (Sigma-Aldrich, St. Louis, MO), 0.14% sodium bicarbonate (NaHCO_3_; Sigma-Aldrich, St. Louis, MO), and 100 U of penicillin and 0.1 µg of streptomycin per mL (Meiji, Tokyo, Japan) at 37 °C in a humidified atmosphere with 5% CO_2_.

### Pteropine orthoreovirus

Pteropine orthoreovirus strain Garut-50 (PRV50G), isolated previously from a greater flying fox (*Pteropus vampyrus*) in Indonesia [22], was propagated in Vero cells in DMEM medium containing 2% FBS and stored at −80°C until use. Virus titration was performed by plaque assay in the Vero 9013 cell line.

### Micafungin

Micafungin sodium for intravenous infusion (Nipro Corporation, Osaka, Japan) was dissolved in sterile DMSO at a concentration of 10 mM to prepare stock solutions and stored at −30°C until use.

### Virus infection, drug treatment, and RNA extraction

A549 cells were seeded in a 6-well plate at a density of 1.5 × 10^6^ cells per well and incubated in DMEM containing 10% FBS for 24 hours to form a monolayer. The medium was removed the following day, and the cells were washed twice with 1X phosphate-buffered saline (PBS). Then, DMEM containing 2% FBS was added to the cells, which were infected with PRV50G at a multiplicity of infection (MOI) of 0.1. At 2 hours postinfection (hpi), the inoculum was removed, and micafungin was added to the cells at a final concentration of 20 µM. DMEM containing 2% FBS was used as a control. All treatments were performed in triplicate. After 6 hours of infection, RNA was extracted using ISOGEN2 (Nippon Gene, Tokyo, Japan). Total RNA was stored at −80°C until used for RNA sequencing.

### RNA-Seq

RNA samples were sent to Macrogen (Kyoto, Japan) for next-generation sequencing. The resulting FASTQ files were analyzed using the supercomputer system at the National Institute of Genetics (Shizuoka, Japan). Quality checks and adapter trimming (using the -g option) were performed using Fastp software (v0.20.1). For mapping, the human reference genome GRCh38.p13 (National Center for Biotechnology Information; NCBI) was used, and gene annotations were obtained using GENCODE v42 [43]. The human reference sequence Homo_sapiens.GRCh38.cdna.all.fa was downloaded from Ensembl. Mapping was performed using HISAT2 software (v2.1.0, with default settings), followed by conversion of the SAM files to BAM format using samtools (v1.7) [44]. Read counts were obtained for humans using featureCounts (v2.0.1). Differentially expressed genes (DEGs) were identified using the DESeq2 package (v1.36.0) in R (v4.3.2) [45]. Gene Ontology (GO) analysis of significantly upregulated genes was performed using the clusterProfiler package (v4.10.0) [46]. A Venn diagram was created using the web-based tool Venny 2.1 (https://bioinfogp.cnb.csic.es/tools/venny/). Protein-protein interactions were analyzed using the STRING database (v12) (https://string-db.org) [47].

### RT-qPCR

All RNA samples were adjusted to a final concentration of 50 ng/µl. A 4-µl aliquot of each RNA sample was mixed with 2 µl of nuclease-free water and incubated at 65 °C for 5 minutes, after which it was placed on ice for at least 5 minutes. A mixture containing 2 µl of 5X RT buffer, 1 µl of 10 mM dNTPs, 0.5 µl of 10 µM Oligo(dT)20, and 0.5 µl of ReverTra Ace (TOYOBO, Osaka, Japan) was added to the RNA solutions, which were then incubated at 42 °C for 60 minutes and 99 °C for 5 minutes. The cDNA was stored at −80°C until required for further analysis. The expression of candidate genes was detected by real-time qPCR using specific primers (Supplementary Information 1B). THUNDERBIRD Probe and SYBR qPCR Mix (TOYOBO, Osaka, Japan), which is optimized for dye-based quantitative PCR (qPCR), was used as the reaction master mix. For cDNA amplification, 1 µl of cDNA was combined with 5 µl of THUNDERBIRD Probe qPCR Mix, 0.5 µl of 10 µM gene-specific forward and reverse primers, and 3 µl of nuclease-free water, adjusted to a final volume of 10 µl. The thermal cycling was performed using a LightCycler System (Basel, Switzerland). Gene expression was analyzed using the 2^-ΔΔCt^ method [48] to determine fold differences.

### siRNA and siRNA transfection

The sequences of the siRNAs used in this study are as follows: siRNA IL-6 sense, 5’-GGACAUGACAACUCAUCUCtt-3’; siRNA IL-6 antisense, 5’-GAGAUGAGUUGUCAUGUCCtt-3’; siRNA scrambled control sense, 5’-UUCUCCGAACGUGUCACGUtt-3’; siRNA scrambled control antisense, 5’-ACGUGACACGUUCGGAGAAtt-3’ (FASMAC, Kanagawa, Japan). Polyethylenimine (PEI) was used as the transfection reagent for knockdown in the cell line, following the protocol of Höbel and Aigner [49]. A549 cells were transfected with 250 pmol of siRNAs for 8 hours, after which the siRNAs were removed. Transfection reagent without siRNAs was used as a control. DMEM containing 2% FBS was added to the transfected cells, which were then incubated for 24 hours. Next, PRV was added to the medium, and at 6 hpi, four randomly selected fields were examined under a light microscope, and syncytia were quantified using ImageJ. Total RNA was extracted from cell pellets and stored at −80°C until used for analysis of gene expression and PRV copy number. Cell supernatants were stored at −80°C and used for measurement of viral titers. All treatments were performed in triplicate.

### Virus detection

Total RNA was extracted from infected cells using ISOGEN2, reverse-transcribed into cDNA, and subjected to qPCR using RNA-direct SYBR Green Realtime PCR Master Mix (TOYOBO, Osaka, Japan) under the following conditions: 90 °C for 30 seconds, 61 °C for 20 minutes, and 95 °C for 30 seconds, followed by 35 cycles of 95 °C for 5 seconds, 55 °C for 10 seconds, and 74 °C for 15 seconds. Melting curve analysis was performed with steps at 95 °C for 10 seconds, 65 °C for 60 seconds, and 97 °C for 1 second. Primer sequences were designed to amplify a region of the S4 segment of PRV50G (forward, 5′-TTGGATCGAATGGTGCTGCT-3′; reverse, 5′-TCGGGAGCAACACCTTTCTC-3′; amplicon size, 159 bp). Ct values were plotted to generate a standard curve, and the relative viral RNA copy number compared to the control was determined by dividing the values for the experimental or control samples by the average value for the control samples. These results were then logarithmically transformed, and the mean and standard deviation were calculated.

### TCID_50_

Vero JCRB9013 cells were seeded in a 96-well cell culture plate at a density of 5 × 10^4^ cells per well and incubated for 24 hours to form a monolayer. The following day, a vial of frozen PRV was thawed at 37 ºC for 90 seconds, and the virus was serially diluted tenfold from 10^−1^ to 10^−10^ in DMEM containing 2% FBS. After dilution, 100 µl of each viral dilution was used to inoculate Vero JCRB9013 cells in eight replicate wells. The last two columns of cells were used as negative controls. The cells were examined daily for a cytopathogenic effect (CPE), and after 1 week of infection, the cells were fixed with 50 µl of 4% formalin (v/v) in 1X PBS. The solution was then discarded, and 1% crystal violet (w/v) in 1X PBS was added to stain live cells. Afterwards, the TCID_50_ was calculated using the Spearman-Kärber method.

### Quantification of syncytium formation

At 6 hpi, four randomly selected fields were photographed using a light microscope at 200x total magnification. All treatments were performed in triplicate. Syncytium formation was quantified using ImageJ software. The thresholds were adjusted by covering the syncytial areas and measuring them to determine their total surface area. Then, the image was converted to a binary format to separate adjacent syncytia that might appear as a single mass. The images were analyzed using the default setting, and the measured values were normalized to the corresponding mock-treated controls to account for background variations. The results are presented as the relative syncytial area normalized to the control.

### Recombinant antibodies against PRV p17 protein

PRV RNA was extracted and converted to cDNA by reverse transcription. The full-length p17 gene was then amplified by RT-PCR using the primers p17_BseRI forward (5’-ACGTGGATGACGACGACAAGATGTCCATCCAGCCTCATCT-3’) and p17_BseRI reverse (5’-ATTTGAGGAGAAGCCCGGACTCAGATCGCGAAGCGCTTAT-3’). The amplified p17 gene was then inserted into the BseRI site of the pET-46 Ek_LIC vector, using an NEBuilder HiFi DNA Assembly Cloning Kit (New England Biolabs, Ipswich, MA) and introduced into NEB 5-alpha chemically competent cells. The plasmid was then extracted using a FavorPrep Plasmid Extraction Mini Kit (FAVORGEN, Taiwan) and introduced by transformation into *Escherichia coli* BL21 (DE3) (Thermo Fisher Scientific, Waltham, MA) for the expression of recombinant proteins. The recombinant clones were cultured on Luria-Bertani (LB) agar containing 50 µg of ampicillin per mL at 37 °C. A single recombinant clone was picked and inoculated into LB broth with the same antibiotics and cultured until the OD_600_ value reached 0.4-0.6. Isopropyl-β-D-thiogalactopyranoside (IPTG) was added to the culture to a final concentration of 0.3 mM, and incubation at 37 °C was continued for 3 hours with vigorous shaking. After induction, the recombinant cells were pelleted by centrifugation, and the cell pellet was resuspended in 20 µl of 1X PBS, 25 µl of 2X SDS-PAGE sample buffer, and 5 µl of β-mercaptoethanol. Next, the cell suspension was boiled for 10 min and then centrifuged at 13,000 rpm at 4 °C for 10 min. The sample was loaded onto a 15% SDS-PAGE gel, and after electrophoresis, the gel was stained with Coomassie brilliant blue staining solution and de-stained in nuclease-free water. For protein purification, proteins were extracted from the cell pellet by sonication on ice for 2 minutes in binding buffer (20 mM sodium phosphate, 0.5 M sodium chloride, and 20 mM imidazole, pH 7.4) and centrifugation at 7,000 rpm for 20 min. The supernatant was collected and filtered through a 0.45-micron polyvinylidene difluoride (PVDF) membrane syringe filter, and the protein was purified using a Cytiva HiTrap Chromatography Column (Marlborough, MA). The column was washed with binding buffer, and the sample was eluted with elution buffer (binding buffer containing 500 mM imidazole). The elution buffer was later replaced with 1X PBS using an Amicon Ultra-15 Centrifugal Filter Unit (Merck Millipore, Darmstadt, Germany). Fractions containing purified protein were pooled and stored at 4 °C. The affinity-purified recombinant p17 protein (50 µg) was mixed with TiterMax (TiterMax USA Inc., Norcross, GA) Gold Adjuvant in a 1:1 ratio in a final volume of 100 µL, and after incubation for 15 minutes at room temperature, the mixture was injected subcutaneously under the back skin of ICR mice. After two weeks (day 14), mice received a booster dose consisting of the same protein mixed with an adjuvant, administered in the same manner as the primary injection. A final booster was administered on day 42 (six weeks after the initial immunization). This final injection consisted of the recombinant p17 protein without any adjuvant to avoid prolonged immune stimulation. This boost was given 3-4 days before blood collection. Blood samples were collected once a week before the final dose. On days 45-46, blood samples were collected from each mouse by cardiac puncture under deep anesthesia, and serum antibody titers were determined by the enzyme-linked immunosorbent assay (ELISA) method. The sera of immunized mice were used for functional viral inhibition assays.

### ELISA

2.5 µg of recombinant p17 protein was coated onto microplates for ELISA and incubated overnight at 4 °C. The next day, the coated wells were washed three times with 100 µl of phosphate-buffered saline with Tween 20 (PBS-T). Then, 5% skim milk was added to block non-specific binding for 1 hour. Serum from immunized mice, diluted 1:10, 1:100, 1:500, and 1:1000, was added to the wells. Serum from non-immunized mice was used as a control. After incubation for 2 hours, the wells were washed three times with PBS-T, and horse anti-mouse IgG antibody (H+L) peroxidase conjugate (Vector Laboratories, Newark, CA) was diluted 1:1000 and added to each well. After incubation for 1 hour, the wells were washed five times with PBS-T. Finally, 100 µl of ABTS 2-Component Microwell Peroxidase Substrate was added to each well. After incubation for 10 minutes, the absorbance at 405 nm was read using an iMark Microplate Absorbance Reader (Bio-Rad Laboratories, Inc., Hercules, CA) to measure the signal from the p17 protein. The serum titer was defined as the highest dilution that still produced a positive test result when compared to normal serum.

### Functional viral inhibition assay

A549 cells were seeded into a 12-well plate at a density of 1 × 10^6^ cells per well and allowed to form a monolayer. The next day, the medium was removed, and the cells were washed twice with 1X PBS. They were then infected with PRV50G at an MOI of 0.01 in DMEM containing 2% FBS. At 2 hpi, the inoculum was removed, and antiserum, MCFG, or a mixture of antiserum and MCFG was added to the cells. Mock-infected and PRV-infected cells were used as controls. All treatments were performed in triplicate. At 6 hours postinfection, random areas of the cell monolayer were imaged under a microscope, and syncytia were quantified using ImageJ. Total RNA was extracted from cell pellets and stored at −80°C until it was used for determination of the PRV copy number and measurement of IL-6 gene expression.

### Statistical analysis

All graphs in this study were created, and statistical analysis of the relative mRNA expression levels and virus copy numbers was performed using the non-parametric *t*-test in Prism 8 software (GraphPad Software Inc., San Diego, CA). A *p*-value of less than 0.05 was considered significantly different. Statistical significance was evaluated as follows: n.s., not significant, *, *p* < 0.05; **, *p* < 0.01; ***, *p* < 0.001; ****, *p* < 0.0001.

## Results

### Identification of p17 as a target of MCFG

**Fig. 1:**
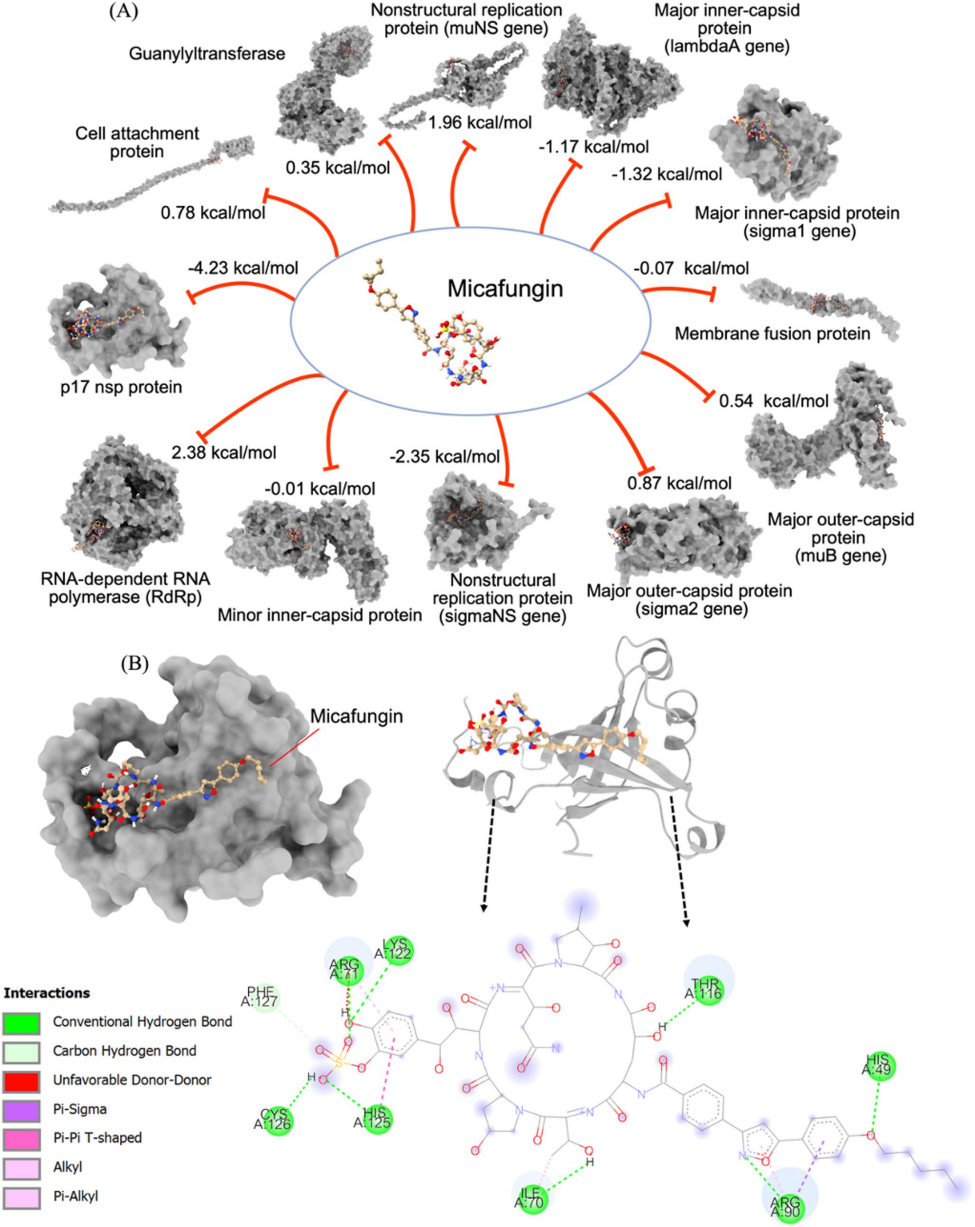
(**A**) Analysis of potential binding interactions of micafungin (MCFG) with twelve PRV target proteins, using AutoDock. (**B**) Docking interactions of MCFG with p17, showing the surface structure of the best binding mode within the protein pocket (left), the 3D structure of amino acid residues involved in the interaction with the MCFG ligand (top right), and a 2D representation of MCFG binding interactions (bottom right). Conventional hydrogen bonds are indicated by dark green dashed lines, carbon-hydrogen bonds are indicated by light green dashed lines

To predict the potential target of micafungin, *in silico* docking was used. The results showed that the non-structural protein p17 was predicted to bind MCFG with the highest efficiency among the 12 PRV proteins tested. p17 demonstrated the most favorable binding energy binding energy (−4.23 kcal/mol), followed by the nonstructural replication protein σNS (−2.35 kcal/mol), the major inner capsid protein σ1 (−1.32 kcal/mol), the major inner capsid protein λA (−1.17 kcal/mol), and the membrane fusion protein (−0.07 kcal/mol) (Fig. 1A). The predicted binding interactions between micafungin and p17 are depicted in Fig. 1B. The amino acid residues engaged in the interaction with the MCFG ligand demonstrated conventional hydrogen bond interactions with His49, Arg90, Ile70, Arg71, Thr116, Lys122, His125, and Cys126, as well as carbon-hydrogen bond interactions with Phe127 (Fig. 1B, right).

### Syncytium formation

**Fig. 2:**
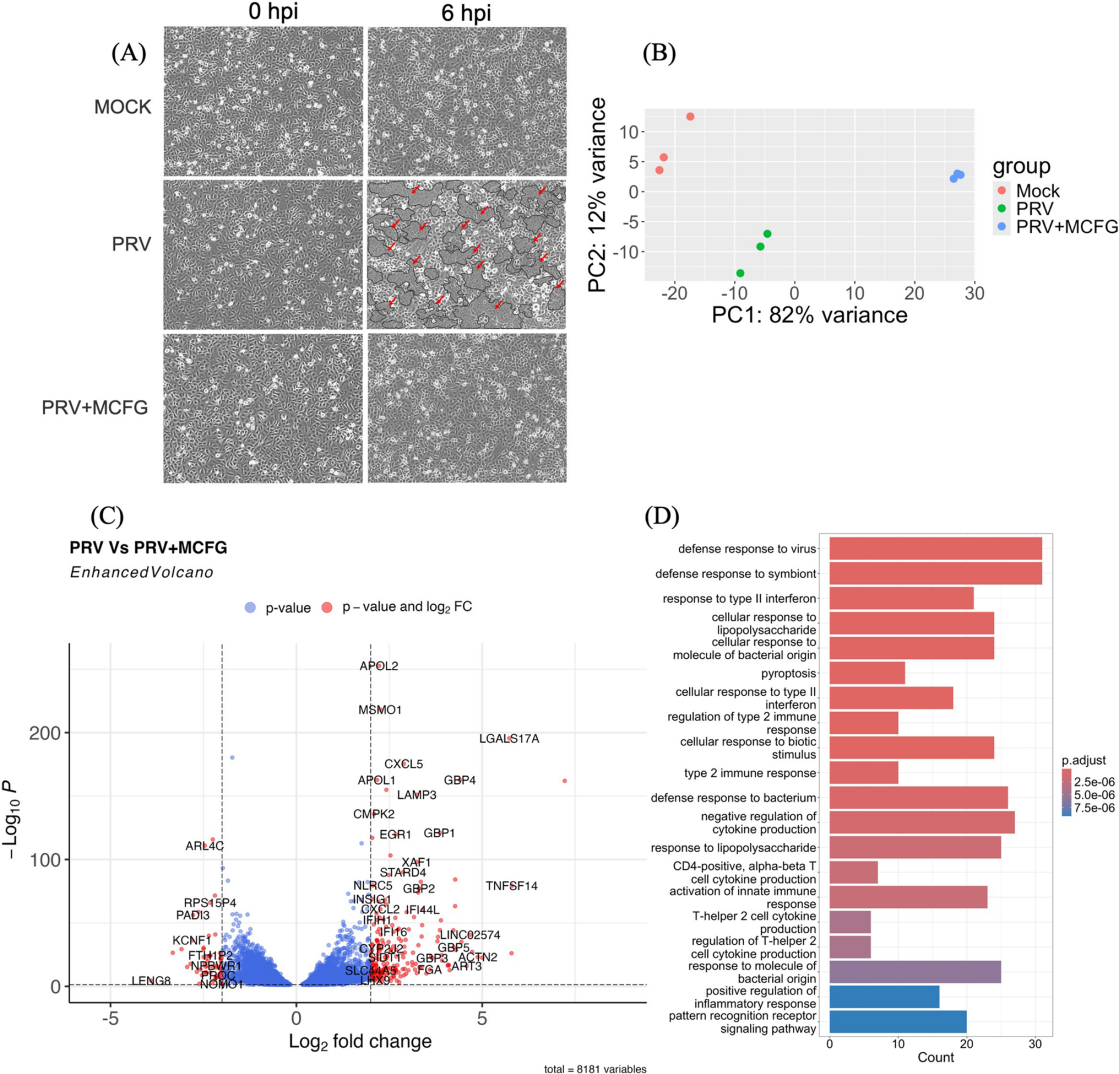
(**A**) Morphology of PRV-infected A549 cells with or without treatment with 20 μM MCFG at 6 hpi. A syncytium formation, indicated by arrows. (**B**) Principal component analysis of RNA-seq data, showing sample-to-sample variation for three replicates. (**C**) Volcano plot depicting differentially expressed genes in PRV-infected A549 cells with or without MCFG treatment. Red dots represent genes with -log_10_P values greater than 0.05 and log2 fold change values greater than 2. (**D**) Gene Ontology (GO) enrichment of DEGs between PRV-infected cells with and without MCFG treatment

Syncytium formation is one of the morphological markers that can be used to evaluate the pathological effect of PRV and the antiviral effect of micafungin. The time course of syncytium formation after PRV infection in A549 cells was examined in order to investigate the effect of micafungin. Syncytia began forming at approximately 6 hpi, and MCFG treatment started to suppress syncytium formation at this time point (Fig. 2A).

### Differential gene expression profiles

Principal component analysis (PCA) was used to examine differential gene expression in mock-infected cells, PRV-infected cells, and PRV-infected cells treated with MCFG (Fig. 2B). Each independent replicate clustered closely within its respective treatment group; however, the mock-infected and micafungin-treated samples exhibited greater dispersion than the PRV-infected samples. Principal component 1 (PC1) represented 82% of the variance, distinguishing the mock-infected and PRV-infected cells from the micafungin-treated cells, while principal component 2 (PC2) represented 12% of the variance between the mock-infected and micafungin-treated cells and the PRV-infected cells. A total of 203 differentially expressed genes (DEGs) were identified between PRV-infected cells and those treated with MCFG, with the indoleamine 2,3-dioxygenase 1 (IDO1) gene exhibiting the most significant upregulation. Conversely, 58 genes were downregulated, with the leukocyte receptor cluster member 8 (LENG8) gene exhibiting the lowest expression level (Supplementary Information 1C). These DEGs are displayed in a volcano plot in Figure 2C. Gene Ontology (GO) enrichment of DEGs was performed for further functional analysis, focusing on the biological process (BP) category. The top 20 enriched BP terms are presented in Figure 2D, with the most significantly enriched terms being"defense response to viruses"and"defense response to symbionts".

**Fig. 3:**
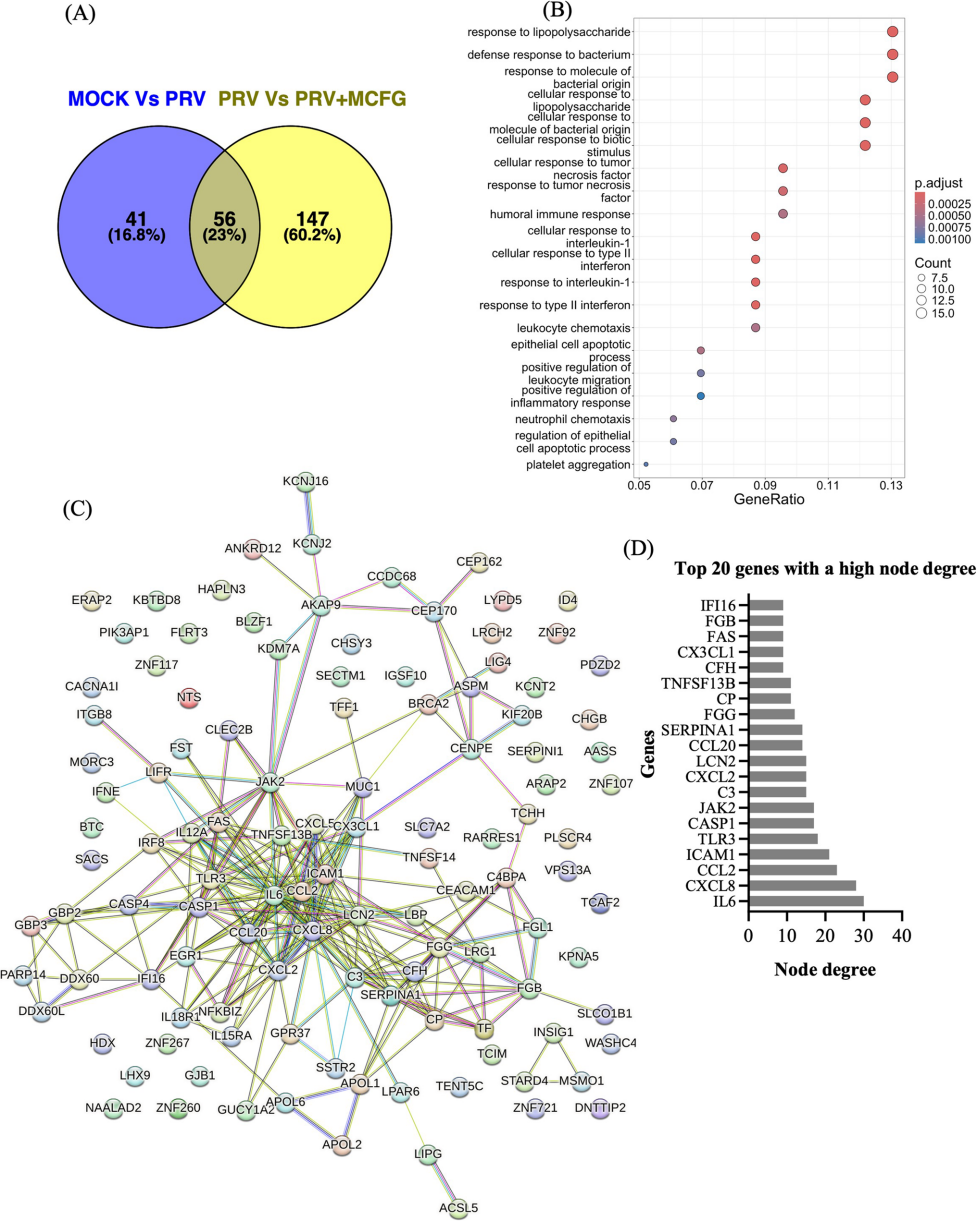
(**A**) Venn diagram showing the differentially upregulated genes between mock-infected and PRV-infected cells with and without MCFG treatment. (**B**) The top 20 enriched biological process pathways with a *p*-value < 0.001. The *x*-axis represents the Z-score value, indicating the degree of enrichment. (**C**) PPIs of the DEGs, identified using the STRING database, representing interacting pathways derived from 147 genes identified across all selected gene lists. (**D**) Bar graph showing the top 10 genes with the highest node degree values from the STRING protein interaction network

A comparative analysis of DEGs was performed to identify genes associated explicitly with MCFG treatment during PRV infection. Venn diagram analysis compared DEGs between mock-treated and PRV-infected cells (MOCK vs. PRV) and between PRV-infected cells and PRV-infected cells treated with MCFG (PRV vs. PRV + MCFG), highlighting genes uniquely expressed in MCFG-treated PRV-infected cells. A total of 147 upregulated genes were identified (Fig. 3A), and the details are provided in Supplementary Information 1D. GO enrichment analysis revealed that the top enriched biological processes among the upregulated genes were"response to lipopolysaccharide"and"defense response to bacterium" (Fig. 3B). Protein-protein interaction (PPI) network analysis (Fig. 3C) identified the interleukin 6 (IL-6) gene as a central hub, exhibiting the largest number of interactions and being closely associated with enriched GO terms (Fig. 3D).

**Fig. 4:**
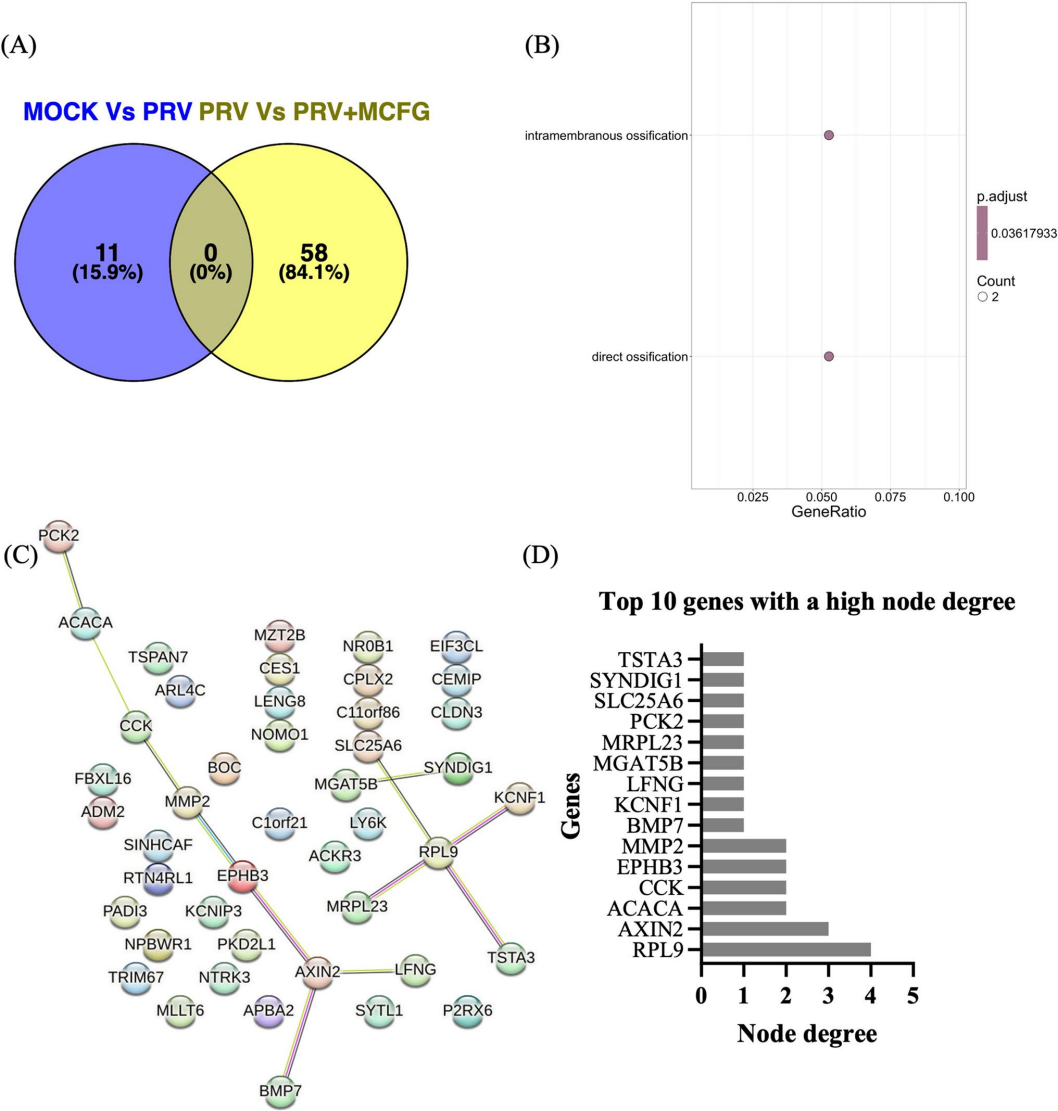
(**A**) Venn diagram showing the differentially downregulated genes between mock-infected and PRV-infected cells, compared to PRV-infected cells with and without MCFG treatment. (**B**) The top enriched biological process pathways with a *p*-value < 0.001. The *x*-axis represents the Z-score value, indicating the degree of enrichment. (**C**) PPIs of the DEGs, identified using the STRING database, representing interaction pathways derived from 58 genes identified across all selected gene lists. (**D**) Bar graph showing the top 20 genes with the highest node degree values from the STRING protein interaction network

A total of 58 unique downregulated genes were identified (Fig. 4A), with further details in Supplementary Information 1D. GO enrichment analysis highlighted"intramembranous ossification"and"direct ossification"as the most enriched biological processes (Fig. 4B). PPI analysis revealed the ribosomal protein L9 (RPL9) gene to be the central node among the downregulated genes, exhibiting the largest number of interactions (Fig. 4C). The top 10 downregulated genes with the largest number of interactions are presented in Figure 4D.

Among the top 20 upregulated genes in MCFG-treated PRV-infected cells, IL-6 was chosen for further examination because of its significant role in the immune response and its pivotal position in the PPI network.

### IL-6 knockdown

**Fig. 5:**
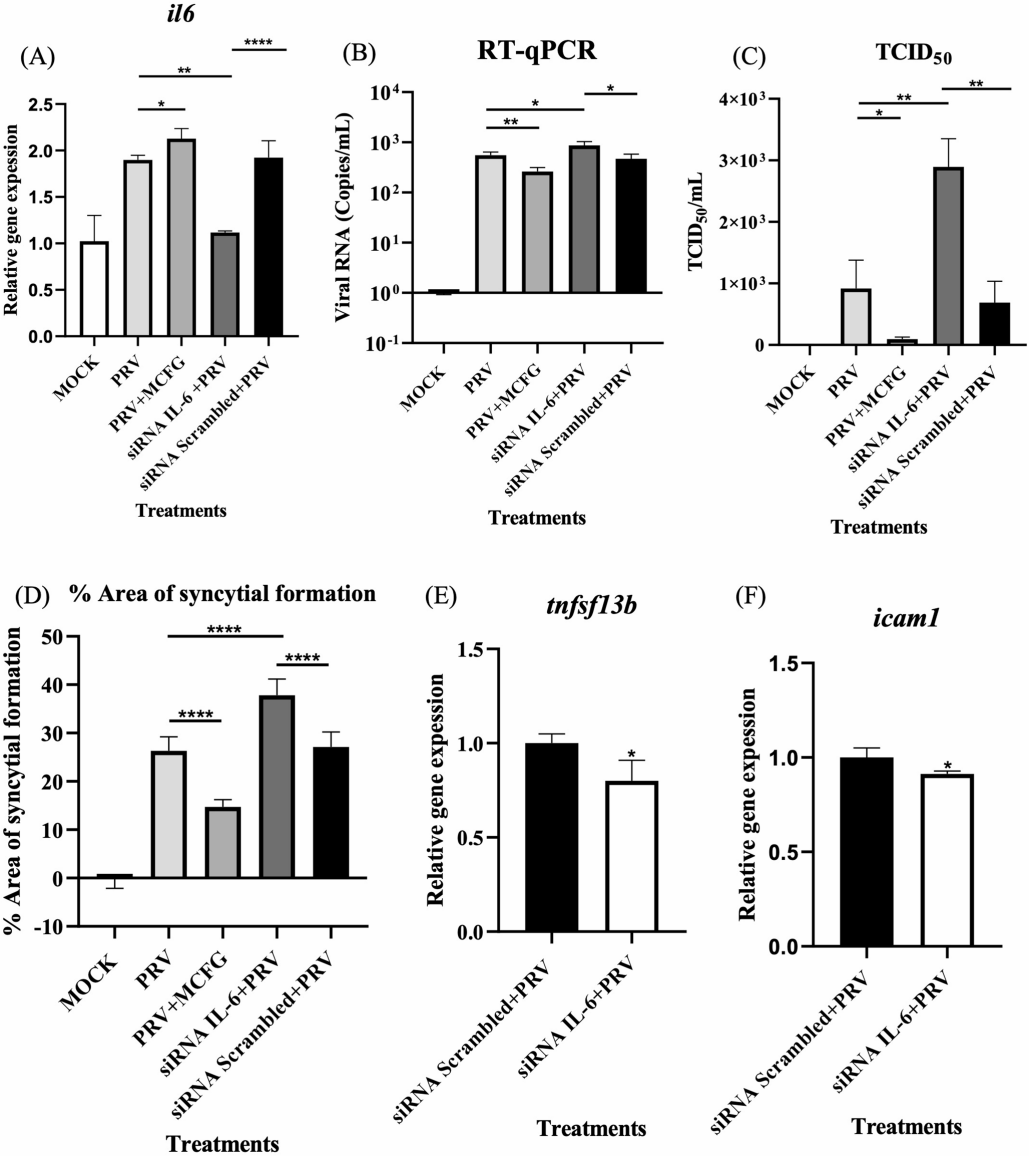
(**A**) Effect of treatment with an siRNA against IL-6 or with MCFG on IL-6 gene expression. Relative expression values were calculated using the 2^-ΔΔCt^ method. The statistical significance of dif- ferences between groups was determined using Student’s *t*-test. Bars represent the mean (*n* = 3) relative gene expression from three inde- pendent experiments with standard deviation (*, *p*-value < 0.05; **, *p* < 0.01; ***, *p* < 0.001; ****, *p* < 0.0001). (**B**) Viral copy numbers determined by qPCR, with data represented as the mean (*n* = 3). (**C**) Viral titer in the supernatant determined by TCID_50_ assay (*n* = 3). (**D**) The percentage of the total area containing syncytia, calculated using ImageJ software (*n* = 12). Effect of treatment with an siRNA against IL-6 on the expression of (**E**) TNFSF13B and (**F**) ICAM1

The combination of PRV infection and MCFG treatment was found to induce IL-6 gene expression at a higher level than PRV infection alone (*p* < 0.05; Fig. 5A). MCFG also slightly inhibited PRV RNA replication (*p* < 0.01; Fig. 5B) while considerably decreasing PRV release (*p* < 0.05; Fig. 5C) and syncytium formation (*p* < 0.0001; Fig. 5D). To investigate the role of IL-6 in PRV propagation, IL-6 gene knockdown was performed by treating cells with an siRNA directed against IL-6 prior to PRV infection, resulting in significant downregulation of IL-6 expression in siRNA-treated cells when compared to PRV-infected cells without siRNA treatment (*p* < 0.01) and those treated with a scrambled siRNA control construct following PRV infection (*p* < 0.0001; Fig. 5A). Viral copy numbers, measured by RT-qPCR, were markedly elevated in IL-6 knockdown cells compared to PRV-infected cells lacking siRNA (*p* < 0.05) or those treated with scrambled siRNA control (*p* < 0.05; Fig. 5B). Similarly, the virus titer in the supernatant, measured using the TCID_50_ assay, was significantly elevated in IL-6 knockdown cells compared to PRV-infected cells (*p* < 0.01) and scrambled siRNA controls (*p* < 0.01; Fig. 5C). In addition, IL-6 knockdown substantially enhanced syncytium formation when compared to PRV-infected cells (*p* < 0.0001) and scrambled siRNA controls (*p* < 0.0001; Fig. 5D). The IL-6 gene was identified as highly interconnected with other genes within the PPI network (Fig. 3C). Thus, the effect of IL-6 knockdown on the expression of related genes was examined. Tumor necrosis factor superfamily member 13B (TNFSF13B) and intercellular adhesion molecule 1 (ICAM1) were slightly downregulated in IL-6 knockdown cells compared to scrambled siRNA-treated cells (*p* < 0.05; Fig. 5E and F).

### Inhibition of p17 protein

**Fig. 6:**
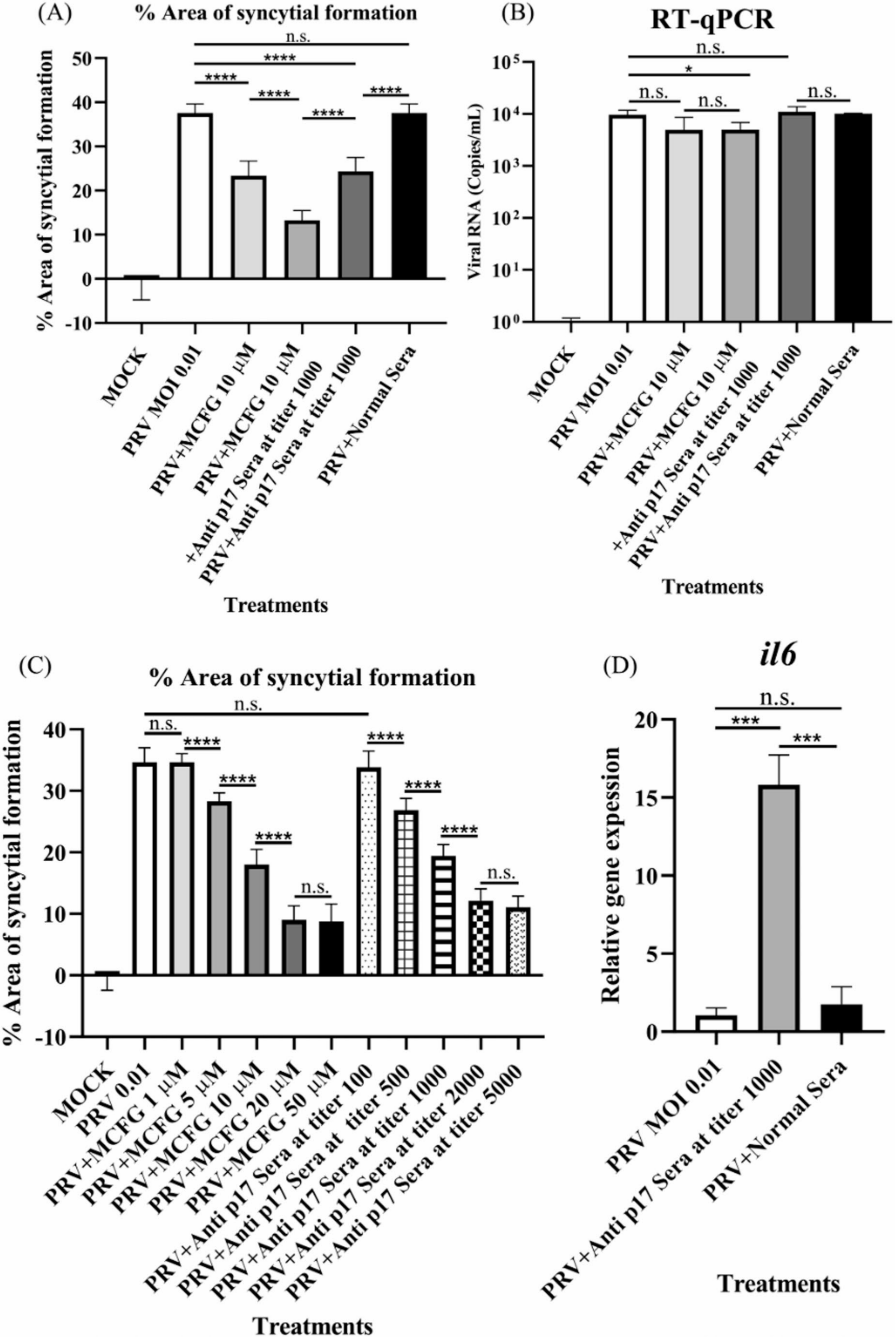
(**A**) Analysis of syncytium formation using ImageJ software (*n* = 12). Statistical significance was determined using Student’s *t*-test (*,* p*-value < 0.05; **, *p* < 0.01; ***, *p* < 0.001; ****, *p* < 0.0001). (**B**) Viral copy numbers determined by qPCR (*n* = 3). (**C**) Percentage of syncytium formation observed after treatment with different concentrations of MCFG or anti-p17 serum (*n* = 12). (**D**) IL-6 gene expression (*n* = 3)

MCFG was found to bind recombinant p17 protein at low does, with saturation occurring at a concentration below 100 nM (Supplementary Information 2A). Treatment of PRV-infected cells with anti-p17 antibody reduced the area of syncytium formation when compared to infected cells treated with normal serum (*p* < 0.0001) or no serum (*p* < 0.0001). The combination of MCFG and the anti-p17 antibody led to a significant decrease in syncytium formation compared to either treatment alone, with *p* < 0.0001 for the drug alone and *p* < 0.0001 for the anti-p17 antibody alone (Fig. 6A). As illustrated in Fig. 6B, the anti-p17 antibody did not substantially influence viral RNA replication. Likewise, both MCFG and anti-p17 treatment resulted in a substantial decrease in syncytium formation in a dose-dependent manner, with 20 µM MCFG and anti-p17 antibody at a titer of 2000 exhibiting the strongest effect (Fig. 6C). The standard curve is shown in Supplementary Information 2B. Expression of the IL-6 gene was significantly upregulated in cells treated with anti-p17 antibody compared to those treated with PRV alone (*p* < 0.001) and the normal serum control (*p* < 0.001), as depicted in Fig. 6D.

## Discussion

Micafungin is an inhibitor of 1,3-β-glucan synthase, an enzyme that plays a crucial role in fungal cell wall synthesis [30]. In addition, MCFG and its analogs, including caspofungin and anidulafungin, have been shown to have antiviral properties against several viruses, including enterovirus 71, chikungunya virus, dengue virus, Zika virus, SARS-CoV-2, and PRV [32–34, 36, 50, 51]. In this study, we investigated the mechanism by which MCFG inhibits PRV propagation in human A549 cells.

IL-6, a pleiotropic cytokine that plays a central role in immune modulation and response to infections, is produced in response to tissue damage and infections. It exerts its effects through the JAK/STAT3 signaling pathway, which influences various immune responses, including inflammation, T-cell differentiation, and tissue repair [52]. IL-6 is particularly important in coordinating pro-inflammatory and anti-inflammatory responses, making it a critical player in controlling viral replication and immune function [53, 54]. In the context of viral infections, IL-6 helps to regulate the immune system by enhancing cytotoxic T-cell activity and IgG production and by promoting tissue repair while reducing virus-induced apoptosis [55]. IL-6 has been shown to play an important role in limiting infections with viruses such as hepatitis B virus (HBV) by inhibiting viral replication and interfering with expression of the viral receptor [56, 57]. PRV infection of the nasopharyngeal cell line NP460 has been shown to downregulate IL-6 cytokine expression, but the mechanism by which this occurs is not known [58]. Our study demonstrates that IL-6 suppresses PRV RNA replication slightly (Fig. 5B) and significantly reduces viral release and syncytium formation in A549 cells, which is similar to the effect of MCFG treatment (Fig. 5C and D). Suppression of the IL-6 gene led to significant down regulation of TNFSF13B and ICAM1 (Figs. 5E and F). These changes suggest that IL-6 might act as a central regulator of the host response to micafungin treatment during PRV infection.

The orthologous nonstructural proteins of other reoviruses, such as ARV p17, BRV p16, and BroV p16, are encoded by the second open reading frame (ORF) of the S1 or S4 gene segment. [8–11, 59]. Previous findings indicate that ARV p17 functions as a CRM1-independent nucleocytoplasmic shuttling protein, possessing a nuclear localization signal defined by the basic amino acids, K122 and R123, which are conserved among ARV strains [60]. ARV p17 has the ability to activate the p53 signaling pathway while inhibiting the PI3K/AKT/mTOR and ERK pathways. These interactions lead to impaired cellular translation, cell cycle arrest, and autophagosome formation, favoring viral replication [61–65]. Specifically, PRV p17 plays an important role in enhancing FAST-mediated cell-cell fusion or syncytium formation, a key process for viral spread in bat cells. However, overexpression of PRV p17 can lead to excessive fusion, resulting in host cell damage. This observation points to the presence of a regulatory mechanism that controls PRV p17 expression, possibly through interactions with the host translation machinery, which may be bat-specific [66]. The addition of anti-p17 antiserum at a titer of 1000 significantly reduced syncytium formation in PRV-infected cells (Fig. 6A), while viral RNA replication remained unaffected (Fig. 6B). This is consistent with previous reports showing that p17 is not essential for PRV replication in A549 cells [66]. The combination of 10 µM MCFG and anti-p17 antiserum at a titer of 1000 had a significantly stronger inhibitory effect on syncytium formation than either treatment alone (Fig. 6A), suggesting that MCFG and anti-p17 antibodies synergistically inhibit the p17 protein, which promotes cell-cell fusion. Syncytium formation was dose-dependently reduced with higher concentrations of MCFG or anti-p17 antiserum. The strongest effect was observed with 20 µM MCFG and anti-p17 antiserum at a titer of 2000 (Fig. 6C). Consequently, specific antibodies targeting p17 increased IL-6 expression in infected host cells compared to those not treated with serum or those treated with normal serum (Fig. 6D). These observations suggest that the suppression of p17 protein induces IL-6 gene expression.

The observation that MCFG treatment, in addition to reducing syncytium formation, has a slight inhibitory effect on PRV RNA replication, whereas the effect of the antibody against p17 is limited to inhibiting syncytium formation suggests that MCFG might also inhibit other processes in the PRV replication cycle. This is supported by our molecular docking study, which showed that MCFG has the potential to bind to the nonstructural replication protein σNS. Previous research has indicated that the σNS protein of orthoreoviruses is essential for viral replication and assembly, functioning as an RNA-binding and stabilizing agent that is crucial for the formation of viral replication factories. Its structural properties enable the compartmentalization of replication machinery, thereby ensuring efficient genome replication and capsid assembly [67]. Furthermore, the observed ability of σNS to stabilize the viral RNA helps to preserve the integrity of the viral replication process [68]. σNS has been shown to assemble into oligomers that associate with viral RNAs and direct them to the organelles where replication occurs. This recruitment function ensures the availability of viral RNAs for transcription and incorporation into new virions [69].

In summary, this study revealed that MCFG potentially targets PRV p17, effectively inhibiting viral release and syncytium formation while slightly reducing PRV replication in A549 cells. Notably, MCFG might target other PRV proteins in addition to p17, such as σNS. Given these findings, MCFG shows promise as an antiviral treatment option for controlling PRV propagation and its associated pathogenesis.

## Supplementary Information

Below is the link to the electronic supplementary material.

Supplementary file1 (XLSX 69.2 KB)

Supplementary file2 (DOCX 177 KB)

## Data Availability

All of the data from this study are included in this article or the supplementary information.
