# Supplementary material for: The mechanism of action of micafungin against pteropine orthoreovirus infection in the human A549 cell line
Source: Arch Virol. 2025 Aug 25;170(9):201. doi: 10.1007/s00705-025-06369-4 (PMC12378174; doi:10.1007/s00705-025-06369-4)
Supplement: Supplementary file 2 — Supplementary file2 (DOCX 177 KB) [file 705_2025_6369_MOESM2_ESM.docx]

**The mechanism of Micafungin action to Pteropine orthoreovirus infection in human A549 cell line**

**Wirayatida Bubphasook^1^, Atsuo Iida^1^ and Eiichi Hondo^1^**

*^1^Laboratory of Animal Morphology, Graduate School of Bioagricultural Sciences, Nagoya University, Nagoya 464-8601, Japan*

^*^Corresponding author: Eiichi Hondo, e-mail: ehondo@nuagr1.agr.nagoya-u.ac.jp

Supplementary Information 2A

**Method of Binding assay**

2.5 µg of p17 recombinant protein was mixed with MCFG at final concentrations of 1 nM, 10 nM, and 100 nM and incubated at room temperature for 2 hours. The mixtures were then coated onto ELISA microplate wells and incubated overnight at 4 °C. The next day, wells were washed three times with 100 µl of PBS-T. To block non-specific binding, 5% skim milk was added and incubated for 1 hour at room temperature. Serum from immunized mice, diluted 1:200, was added to each well and incubated for 2 hours. As a control, p17 protein mixed with DMSO was used in place of MCFG. After serum incubation, wells were washed three times with PBS-T. A horseradish peroxidase (HRP)-conjugated Horse Anti-Mouse IgG (H+L) antibody (1:1000 dilution in blocking buffer) was then added and incubated for 1 hour. Following this, the wells were washed five times with PBS-T. To detect the signal, 100 µl of ABTS® 2-Component Microwell Peroxidase Substrate was added to each well and incubated for 10 minutes. The absorbance at 405 nm was measured using an iMark™ Microplate Absorbance Reader to assess the binding signal of p17.

**Results of binding assay**


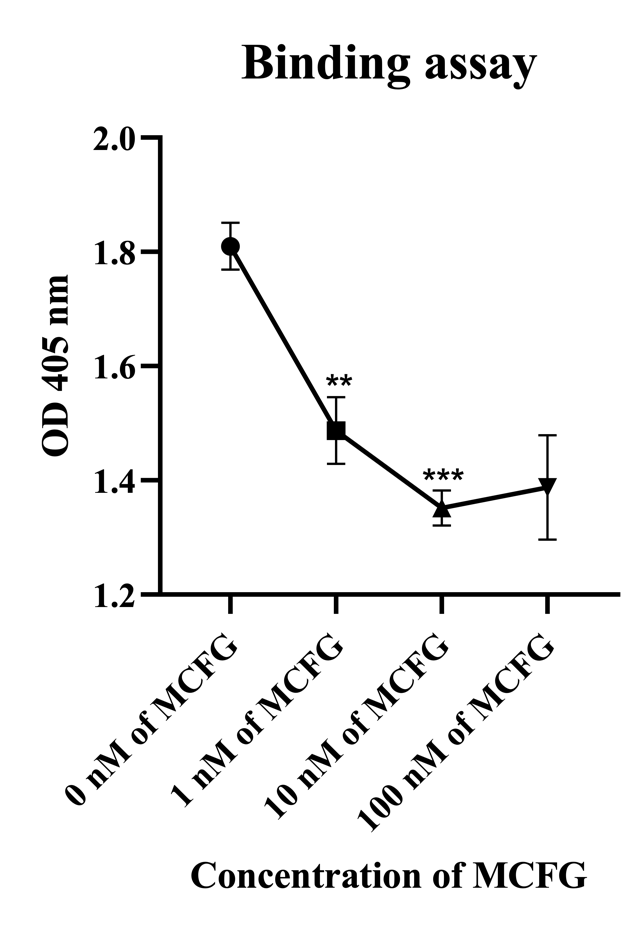


**Fig. 1** Binding assay by ELISA (n=3).

Supplementary Information 2B


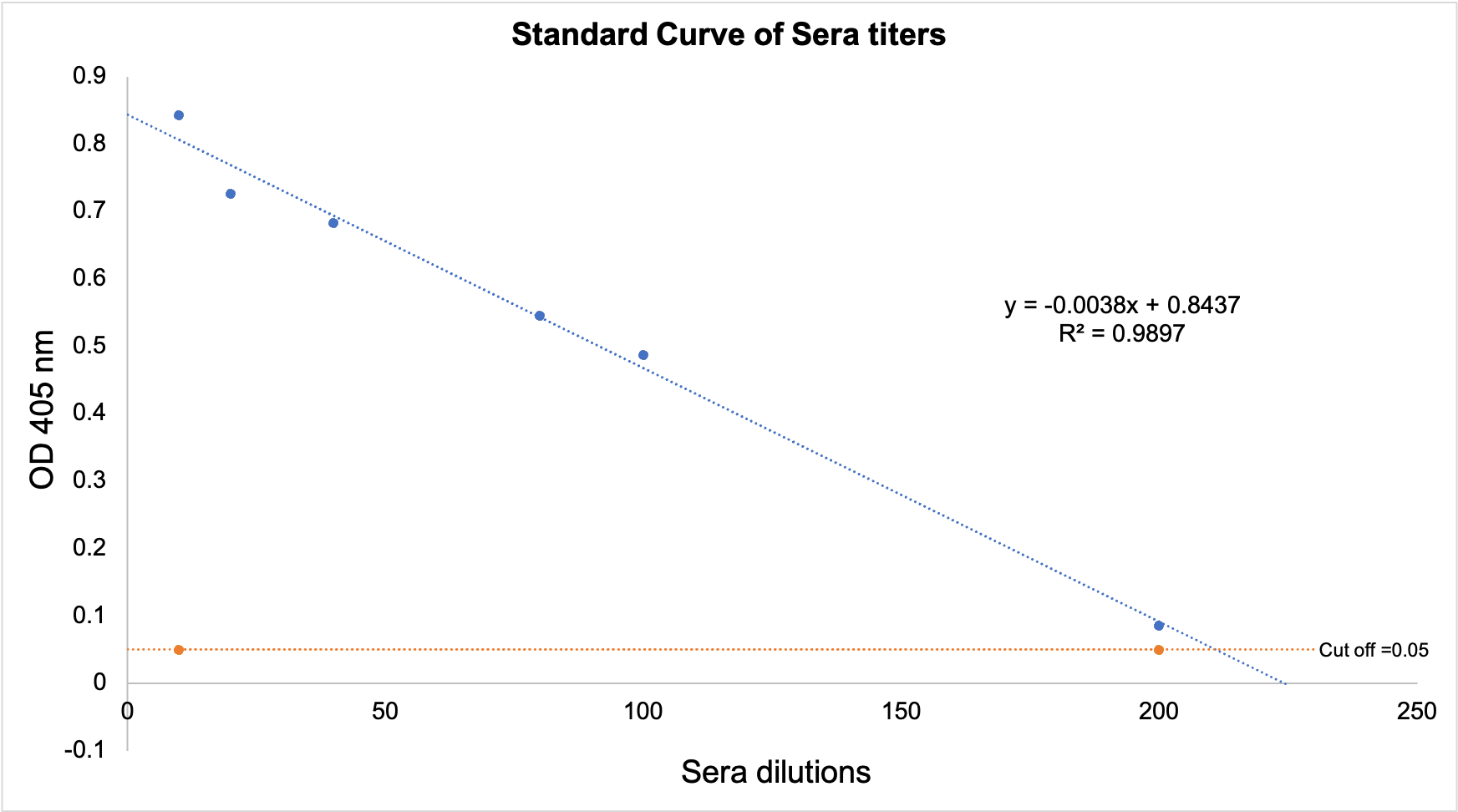


**Fig. 1** The standard curve of sera titers used to evaluate antibody responses in this study.
